# Cardioprotective effects of melatonin against myocardial ischaemia/reperfusion injury: Activation of AMPK/Nrf2 pathway

**DOI:** 10.1111/jcmm.16691

**Published:** 2021-06-14

**Authors:** Chennian Xu, Jian Wang, Zhenge Fan, Shuang Zhang, Rui Qiao, Yu Liu, Jian Yang, Lifang Yang, Huishan Wang

**Affiliations:** ^1^ Department of Cardiovascular Surgery General Hospital of Northern Theater Command Shenyang China; ^2^ Department of Ultrasound Chinese PLA 985 Hospital Taiyuan China; ^3^ Department of Cardiovascular Surgery Xijing Hospital Air Force Medical University Xi'an China; ^4^ Department of Anesthesiology Xi'an Children's Hospital Xi'an China

**Keywords:** AMPK/Nrf2, heart, ischaemia/reperfusion injury, melatonin, oxidative stress

## Abstract

Although reperfusion is the most effective therapy for patients with acute myocardial infarction, reperfusion injury limits the therapeutic effects of early reperfusion. Oxidative stress plays a crucial role in myocardial ischaemia/reperfusion (I/R) injury. Melatonin, a circulating hormone, is well‐known as an antioxidant in cardiovascular diseases. In this short communication, we show that melatonin significantly improves post‐ischaemic cardiac function, reduces infarct size and decreases oxidative stress. Furthermore, melatonin markedly increases AMPK activation and Nrf2 nuclear translocation. Nevertheless, these melatonin‐induced changes are abrogated by compound C. In addition, ML‐385, an Nrf2 inhibitor, also withdraws the antioxidative effects of melatonin but has little effect on AMPK activation. In conclusion, our results demonstrate that melatonin alleviates myocardial I/R injury by inhibiting oxidative stress via the AMPK/Nrf2 signalling pathway.

## 1 INTRODUCTION

Ischaemic heart disease is the leading cause of morbidity and mortality in the United States and other parts of the world.^1^ Reperfusion is the major treatment of choice for preserving left ventricular function and limiting myocardial infarct size.^2^, ^3^ However, the process of restoring coronary blood flow to the ischaemic cardiac tissue can, in itself, induce myocardial injury and cardiomyocyte death, a phenomenon termed ‘ischaemia/reperfusion (I/R) injury.^4^, ^5^ Oxidative stress is considered to be the key pathogenic mechanism of myocardial I/R injury.^6^, ^7^ Therefore, illustrating the molecular mechanisms that could inhibit oxidative stress may be useful to prevent myocardial I/R injury.

Adenosine monophosphate–activated protein kinase (AMPK) has been identified as a pivotal energy sensor and regulator of cellular metabolism that has an important role in the regulation of energy homoeostasis under normal and ischaemic conditions.^8^, ^9^ Nuclear factor erythroid 2–related factor (Nrf2) is an important transcription factor that translocates into the nucleus and controls the expression of many target genes.^10^ Melatonin (Mel) is well‐known for its antioxidant capacity by scavenging free radicals and activating the antioxidant enzymes in a variety of cardiovascular diseases. The present study was aimed to determine the protective actions of Mel against myocardial I/R injury and elaborate whether AMPK/Nrf2 signalling is associated with Mel‐induced cardioprotective effects.

## 2 MATERIALS AND METHODS

### 2.1 Myocardial I/R protocol

Male C57BL/6 mice (8 weeks old, 20‐25 g body weight), were provided by the Laboratory Animal Center at AIR Force Medical University. The myocardial I/R injury model was established as described previously.^11^ Briefly, mice were anaesthetized and ventilated via tracheal intubation with a Harvard rodent respirator. The left anterior descending coronary artery was ligated by placing a 6‐0 silk suture and making a slip knot. Myocardial I/R injury was inflicted by ischaemia for 30 minutes, following by reperfusion for 2 hours or 24 hours. Mel (20 mg/kg) was intraperitoneally injected 10 minutes prior to reperfusion. Compound C (0.25 mg/kg) and ML‐385 (30 mg/kg) were intraperitoneally injected 15 minutes prior to reperfusion.

### 2.2 Echocardiographic measurements

As described in a previous study,^12^ heart function was measured by transthoracic echocardiography with a VisualSonics Vevo 770 echocardiography machine. Briefly, mice were anaesthetized with 1% isoflurane and placed on a thermostatic pad. The left ventricular ejection fraction (LVEF) and left ventricular fractional shortening (LVFS) were measured at the level of the papillary muscles by motion (M‐)–mode echocardiography.

### 2.3 Measurements of myocardial infarct sizes

As described previously,^11^ at the end of reperfusion, the left anterior descending coronary artery was reoccluded, and 2% Evans Blue dye was injected into the left ventricular cavity. Then, the hearts were quickly frozen at −80°C for 1 hours and sectioned horizontally into six slices. The slices were incubated in 1% TTC at 37°C for 15 minutes in the dark and subsequently fixed in 4% paraformaldehyde overnight. Myocardial infarct size was determined using Image‐Pro Plus software (Media Cybernetics).

### 2.4 Determination of SOD activity and MDA content

The activity of SOD and the content of MDA in cardiac tissues were determined using ELISA kits from the Institute of Nanjing Jiancheng Bioengineering Institute.

### 2.5 Western blot analysis

Left ventricular tissue lysates were prepared using RIPA lysis buffer (Thermo Fisher Scientific) containing 1% protease inhibitor cocktail and 1% phosphatase inhibitor cocktail. Denatured samples were subjected to SDS‐polyacrylamide gels and transferred onto PVDF membranes. The membranes were incubated with primary antibodies against phospho‐AMPK (Abcam), AMPK (Abcam), Nrf2 (Abcam), histone H3 (Cell Signaling Technology) and GAPDH (Cell Signaling Technology), followed by horseradish peroxidase–conjugated secondary antibody for 1 hour at room temperature, as described previously.

### 2.6 Statistical analysis

All data were expressed as mean ± SEM. The statistical significance of differences was determined by the Student *t* test between two groups or one‐way ANOVA, followed by Bonferroni's multiple‐comparison post hoc *t* test. A value of *P* < .05 was considered to be statistically significant.

## 3 RESULTS

### 3.1 Compound C abolished Mel‐induced alleviation of infarct size, cardiac function and AMPK/Nrf2 pathway during myocardial I/R

**FIGURE 1:**
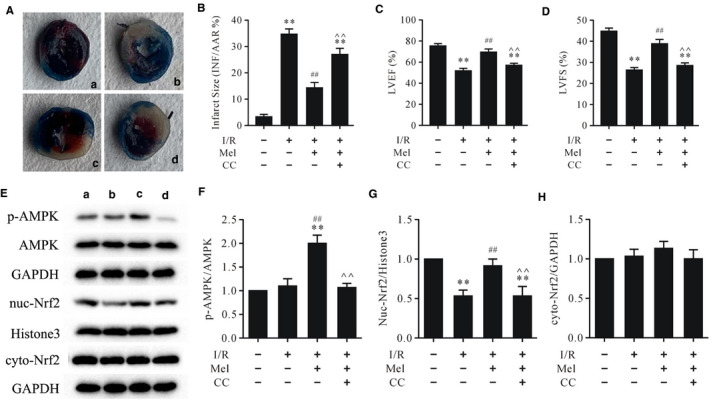
Compound C abrogated the cardioprotective effects of Mel and Mel‐induced AMPK activation and Nrf2 nuclear translocation in myocardial I/R A. Evans Blue and TTC staining of heart slices: A. sham; B. I/R; C. Mel + I/R; d. Mel + CC + I/R; B. myocardial infarct size; C. LVEF; D. LVFS; E. representative immunoblots of p‐AMPK, AMPK, nuc‐Nrf2, histone3 (internal control), cyto‐Nrf2 and GAPDH (internal control); F‐H. Semi‐quantitative analysis of p‐AMPK, nuc‐Nrf2 and cyto‐Nrf2. Data are presented as mean ± SEM, n = 6. TTC: triphenyltetrazolium chloride; LVEF: left ventricular ejection fraction; LVFS: left ventricular fraction shortening; I/R: ischaemia/reperfusion; Mel: melatonin; CC: compound C. ^**^ *P* < .01 vs. the sham group, ^##^ *P* < .01 vs. I/R group, ^^*P* < .01 vs. the Mel + I/R group

As revealed in Figure 1, I/R treatment dramatically increased infarct size and decreased LVEF and LVFS. Moreover, I/R treatment could significantly reduce the protein level of intranuclear Nrf2 but had little effect on the protein levels of p‐AMPK and cytoplasmic Nrf2. In particular, compound C could significantly obstruct the cardioprotective effects and activation of the AMPK/Nrf2 pathway of Mel in myocardial I/R.

### 3.2 ML‐385 attenuated antioxidative effects and Nrf2 nuclear translocation but had little effect on AMPK activation in Mel‐treated myocardial I/R

**FIGURE 2:**
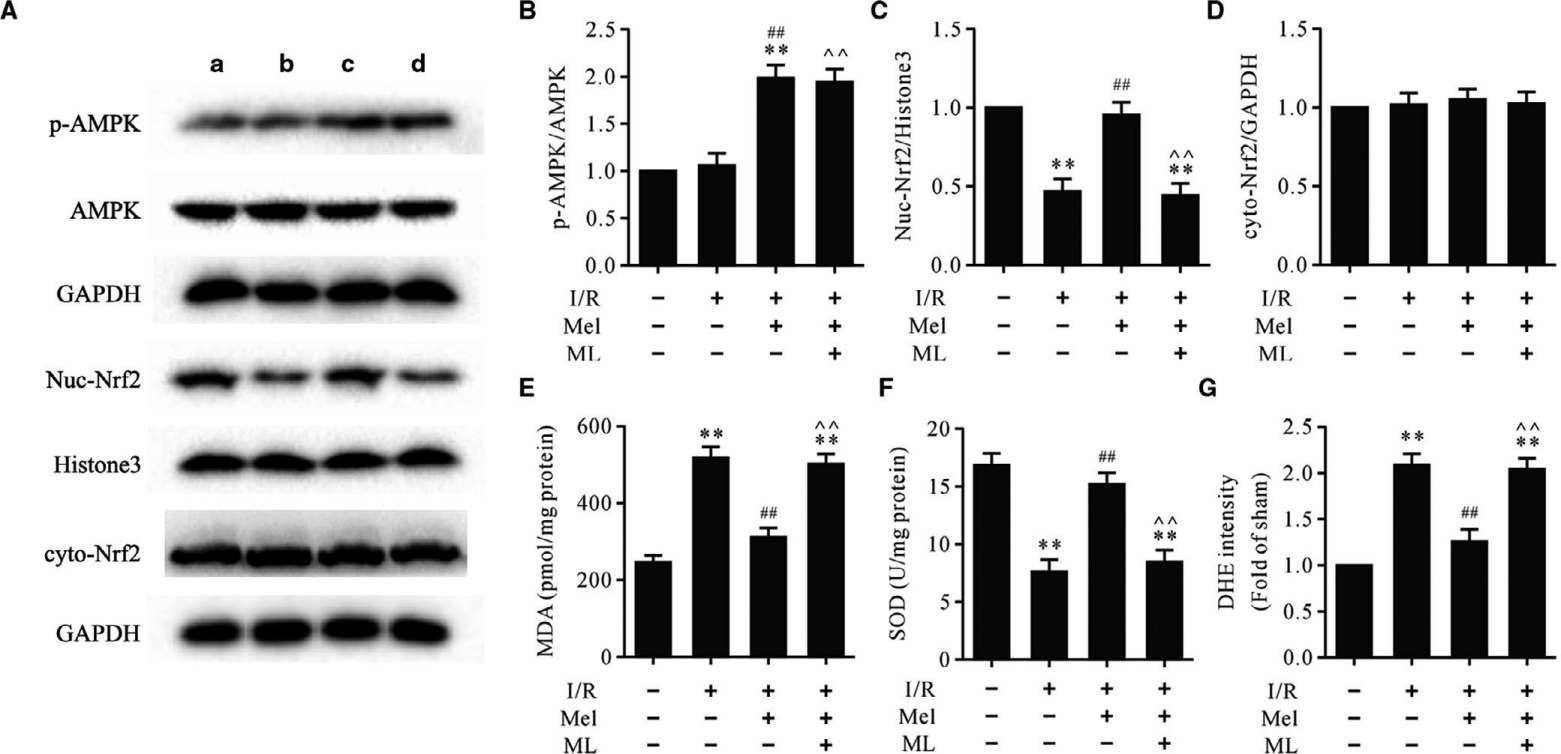
ML‐385 retarded Mel‐induced Nrf2 nuclear translocation and antioxidative effects but had little effect on AMPK activation in myocardial I/R injury. A. Representative immunoblots of p‐AMPK, AMPK, nuc‐Nrf2, Histone3 (internal control), cyto‐Nrf2 and GAPDH (internal control): A. sham; B. I/R; C. Mel + I/R; D. Mel + CC + I/R; B‐D. Semi‐quantitative analysis of p‐AMPK, nuc‐Nrf2 and cyto‐Nrf2; E. MDA contents; F. SOD activity; G. DHE intensity. Data are presented as mean ± SEM, n = 6. I/R: ischaemia/reperfusion; Mel: melatonin; ML: ML‐385; nuc‐Nrf2: intranuclear Nrf2; cyto‐Nrf2: cytoplasmic Nrf2. ^**^ *P* < .01 vs. the sham group, ^##^ *P* < .01 vs. the I/R group, ^^*P* < .01 vs. the Mel + I/R group

As shown in Figure 2, Western blot and ELISA showed that treatment with Mel increased the protein levels of p‐AMPK, intranuclear Nrf2 and the activity of SOD and decreased the levels of MDA and ROS. However, ML‐385 could reverse Nrf2 nuclear translocation and the levels of MDA, ROS and SOD induced by Mel but had little effect on the protein level of p‐AMPK. These data indicated that AMPK functioned upstream of Nrf2 signalling in Mel‐mediated cardioprotective effects against myocardial I/R.

## 4 DISCUSSION

In the present study, we further demonstrated that the cardioprotective effects of Mel against myocardial I/R injury were associated with inhibiting oxidative stress. More importantly, compound C blocked AMPK activation and Nrf2 nuclear translocation and inhibited the antioxidative effects of Mel. In addition, ML‐385 abolished Nrf2 nuclear translocation and the antioxidative effects but had little effect on AMPK activation.

AMPK functions as a cardiac energy sensor and maintains energy homoeostasis in physiological and pathological conditions.^8^ Our results demonstrated that I/R treatment with Mel significantly increased the phosphorylation of AMPK, along with increasing levels of intranuclear Nrf2. However, compound C could frustrate Mel‐dependent regulation of AMPK and Nrf2 in myocardial I/R. Intriguingly, ML‐385 could also thwart Mel‐dependent regulation of Nrf2 but had little effect on AMPK activation, suggesting that Nrf2 acts downstream of AMPK signalling in Mel‐induced cardioprotective effects against myocardial I/R injury.

Oxidative stress is a prominent pathological feature and plays an important role in the pathogenesis of myocardial I/R injury.^13^, ^14^ In the current study, the results showed that the levels of MDA and ROS significantly increased in the I/R group, and SOD activity decreased compared to the sham group. As expected, I/R treatment with Mel decreased the levels of MDA and ROS and improved SOD activity. Nevertheless, ML‐385 could obstruct Mel‐induced antioxidative effects in myocardial I/R injury, suggesting that the antioxidative effects of Mel were related to AMPK and Nrf2. Taken together, this short communication has indicated that the AMPK/Nrf2 signalling pathway plays a pivotal role in mediating the cardioprotective effects of Mel in myocardial I/R injury.

## CONFLICTS OF INTEREST

The authors confirm that there are no conflicts of interest.

## AUTHOR CONTRIBUTION

**Chennian Xu:** Conceptualization (equal); Data curation (equal); Formal analysis (equal); Resources (equal); Writing‐original draft (equal); Writing‐review & editing (equal). **Jian Wang:** Data curation (equal); Formal analysis (equal); Writing‐original draft (equal). **Zhenge Fan:** Conceptualization (equal); Data curation (equal). **Shuang Zhang:** Formal analysis (equal); Supervision (equal). **Rui Qiao:** Data curation (equal). **Yu Liu:** Funding acquisition (equal). **Jian Yang:** Funding acquisition (equal); Project administration (equal); Resources (equal); Writing‐review & editing (equal). **Lifang Yang:** Funding acquisition (equal); Project administration (equal); Supervision (equal); Writing‐review & editing (equal). **huishan wang:** Conceptualization (equal); Funding acquisition (equal); Project administration (equal); Supervision (equal); Writing‐review & editing (equal).

## Data Availability

The data used in this study are available from the corresponding author upon request.
